# Health Disparities Among Mid-to-Older Deaf LGBTQ Adults Compared with Mid-to-Older Deaf Non-LGBTQ Adults in the United States

**DOI:** 10.1089/heq.2019.0009

**Published:** 2019-10-30

**Authors:** Poorna Kushalnagar, Cara A. Miller

**Affiliations:** Department of Psychology, Gallaudet University, Washington, District of Columbia.

**Keywords:** deaf, sign language, deafness

## Abstract

**Purpose:** To compare chronic health and mental health conditions between mid-to-older deaf lesbian, gay, bisexual, transgender, and queer (LGBTQ) and mid-to-older non-LGBTQ adults who are 45 years or older.

**Methods:** Medical conditions and mental health disorders data were gathered from 981 mid-to-older deaf adults (178 LGBTQ and 803 non-LGBTQ) who took the Health Information National Trends Survey in American Sign Language between 2015 and 2019. Modified Poisson regression with robust standard errors was used to calculate relative risk estimates and 95% confidence intervals for all medical conditions and mental health disorders with self-reported LGBTQ status as the main predictor, adjusting for known health correlates.

**Results:** Consistent with the LGBTQ health disparity in the general population, our study findings indicated health disparities for certain medical conditions (e.g., lung disease, arthritis, and comorbidity) and mental health disorders (e.g., depression and anxiety) among mid-to-older deaf LGBTQ compared with non-LGBTQ deaf adults.

**Conclusion:** Like the LGBTQ counterparts in the general population, deaf LGBTQ adults may require more frequent and comprehensive health care services. Culturally and linguistically competent care by providers may be invaluable in reducing such health inequities, particularly when provider education and training is undertaken through an intersectional framework that considers the interaction and context of multiple patient and provider social identities.

## Introduction

The U.S. Healthy People initiative includes plans to reduce health disparities among lesbian, gay, bisexual, transgender, and queer (LGBTQ) individuals by the end of 2030.^1^ Although no research has examined health disparities among sexual and gender minority individuals within the deaf and hard of hearing community (termed as “Deaf” henceforth to reflect a linguistic minority group), there are reports of health disparity between LGBTQ and non-LGBTQ individuals in the general population.^2,3^

## Background

Many Deaf adults who use American Sign Language (ASL) experience barriers to accessing health information and health care, which places them at health knowledge disparity compared with the predominantly hearing, primarily English-speaking population.^4–7^ Nonconcordant patient–physician communication, inaccessible health information, and few qualified sign language interpreters in health care settings are among the barriers encountered by this medically underserved group. In a national study of over 1500 Deaf adults, the prevalence of self-reported hypertension diagnosis was similar for age and gender compared with the general population whereas this differed for race between Deaf and hearing populations.^8^ The authors attributed this racial disparity to the underdiagnosis of hypertension in African American Deaf patients due to possible nonconcordant patient–physician communication and mistrust between such patients and their providers.

Providers who lack or rate low in cultural competence in working with Deaf patients may further perpetuate community and individual histories of inadequate care and poor treatment adherence.^9,10^ In a 2017 smoking and lung disease study of 188 Deaf adults aged 55 to 80 years, Deaf patients who did not experience accessible communication with their doctors were significantly less likely to ask about lung cancer screening testing compared with Deaf patients who used ASL or other sign language interpreters to communicate with their health care providers.^6^ However, in another national study of nearly 300 LGBTQ-identified Deaf adults, the presence of a sign language interpreter in the room did not appear to increase patients' disclosure of their marginalized sexual orientation and/or gender identities (SOGIs) to health care providers.^11^ Instead, this study found that Deaf LGBTQ patients' willingness or hesitation to disclose SOGI was significantly associated with their perceptions of providers' patient–centered communication behavior. This finding suggests that societal stigmas associated with LGBTQ identities may be particularly impactful or salient among LGBTQ Deaf adult patients. Given this, together with extensive discussion about minority stress faced by LGBTQ adults and older adults,^3,12,13^ it may be that mid-to-older LGBTQ Deaf adults may have poorer health outcomes compared with their Deaf non-LGBTQ (heterosexual, cisgender) counterparts.

Among mid-to-older hearing LGBTQ populations, compared with hearing, non-LGBTQ same-age populations, LGBTQ individuals experienced health disparity in terms of physical health. Lesbian, gay, and bisexual adults aged 50 years or older were significantly more likely to report chronic medical conditions than their heterosexual counterparts; similarly, gay and bisexual older men were more likely to report having angina pectoris or cancer than heterosexual older men.^2^ Adults over 50 years who self-identified as transgender, lesbian, or as bisexual women were more likely to report indicators of poorer physical health such as obesity and lower rates of physical activity compared with their non-LGBTQ counterparts.^14–16^ Additionally, chronic conditions were more frequently identified among transgender adults compared with cisgender counterparts, including in particular asthma, chronic obstructive pulmonary disease, depression, and HIV.

Similar disparity has been reported relative to mental health. Self-reported percentages of disability and mental distress were higher among lesbian, gay, and bisexual older adults,^2^ while older transgender adults reported significantly higher depression symptomatology than that reported by their similar-aged cisgender counterparts.^14^ Clearly, significant health-related disparities exist for LGBTQ individuals in the general population and in Deaf individuals; however, not much is known about the health disparity experienced by Deaf individuals who self-identify as LGBTQ.

To date, no available literature has examined Deaf LGBTQ health factors. The little research available on the Deaf older adult population is that those individuals who predominantly use ASL and have lower English literacy typically have gaps in their health-related knowledge along with insufficient health literacy.^5^ As older hearing LGBTQ adults are found to be at greater risk for chronic diseases, depression, and worse physical health than non-LGBTQ counterparts, a similar disparity may exist between Deaf LGBTQ older adults and their Deaf non-LGBTQ same-aged counterparts. Thus, this article aims to explore whether within-group disparity exists for medical conditions among Deaf LGBTQ older adults in comparison to their Deaf non-LGBTQ counterparts.

## Methods

### Survey items and data source

Health Information National Trends Survey (HINTS; hints.cancer.gov) is a survey focused on collecting information about the American public's use of cancer-related information and health communication. HINTS was culturally adapted and translated into ASL. Items were translated and back-translated by Deaf bilingual professionals. The translated measure was then tested for clarity and understanding through cognitive interviews with Deaf people who had a high school education or less schooling. The finalized translation was then filmed and included in an online survey that was administered to a U.S. sample of Deaf adults who used ASL.

Items used in the analysis included sociodemographic variables and medical conditions. Self-reported medical conditions were assessed with this question: *Has a doctor or other health professional ever told you that you had any of the following medical conditions:* Depression or anxiety disorder, cancer, diabetes, hypertension, cardiovascular diseases, chronic lung disease/asthma/emphysema/chronic bronchitis, arthritis/rheumatism, cirrhosis/liver/kidney problems, or stroke.

### Procedure

With approval from the Institutional Review Board, data were collected through the HINTS-ASL between October 2015 and April 2018. Purposive sampling was used to ensure adequate representation of Deaf signers across the United States, including Hawaii and Alaska, with respect to key demographic characteristics including age, education, race, ethnicity, gender identity, and sexual orientation. Recruitment methods included snowball sampling through personal networks, distribution of flyers, and advertisements on Deaf-centered organizations' websites and e-newsletters. Communication between the research staff and participants occurred through accessible channels, including mail, e-mail, social media, and video chat programs.

Prospective participants were provided with an informational flyer and given the opportunity to learn about and discuss the study's purpose and procedures, review criteria for inclusion and exclusion, and pose further questions regarding eligibility and interest. To maximize recruitment of hard-to-reach Deaf LGBTQ individuals, we used relationship-building approaches including making personal contacts and explaining the study in depth before sending informed consent forms. This process frequently necessitated multiple steps of contact before prospective participants agreed to review the informed consent form in ASL and English.

Only those who self-reported using ASL as their primary language were included; exclusion criteria included being under the age of 18 years or reporting unilateral hearing loss. The survey took ∼1 h to complete. Each participant received a $25 gift card for participating in the study. No names or identifying information were included in the online survey, and a unique identifier was used to avoid storing personal information in the same online survey dataset. The identifying information was stored in a separate database that was accessible only to the principal investigator.

### Statistical analyses

Using age-weighted data, descriptive statistics were used to summarize the sample characteristics of mid-to-older Deaf ASL users identifying as non-LGBTQ (*n*=803) and LGBTQ (*n*=178), all who were between 45 and 95 years and had answered the medical condition questions, whether they had medical conditions or not. Comorbidity status was assigned if the respondent reported that they had two or more medical conditions as diagnosed by their health care providers. Modified Poisson regression with robust standard errors was used to calculate relative risk estimates and 95% confidence intervals for all medical conditions with self-reported LGBTQ status as the main predictor, adjusting for known correlates of medical conditions (age, gender, race, education, and health status). The modified Poisson approach is recommended for models with binomial outcomes, and the application of robust standard errors helps rectify the overestimation for the relative risk of having a medical condition.^17^ The adjusted risk ratios were used to estimate the likelihood of having had a medical condition as diagnosed by a health care provider or health professional compared with not having a medical condition (reference category). Data analyses were conducted using SPSS version 25.

## Results

Table 1 presents the sociodemographic characteristics and Table 2 presents the health characteristics for 803 non-LGBTQ Deaf individuals, compared with 178 LGBTQ Deaf individuals, all aged between 45 to 95 years. As seen in Table 1, Deaf LGBTQ participants were significantly more likely to be younger (mean age=58; standard deviation [SD]=9) compared with Deaf non-LGBTQ individuals (mean age=63; SD=11). No group differences were observed for sex, race/ethnicity, education, and body mass index.

**Table 1.** Deaf Sample Sociodemographic and Health Behavior Characteristics

	Non-LGBTQ (*n*=803)		LGBTQ (*n*=178)		*t* (*p*-value)
	Mean	SD	Mean	SD	
Age	63	11	58	9	**5.69 (<0.001)**
BMI	29	6	29	6	−1.37 (=0.17)

	*n* (%)	*n* (%)	*χ^2^* (*p*-value)		
Sex			1.03 (0.31)		
Male	301 (37.5)	74 (41.6)			
Female	502 (62.5)	104 (58.4)			
Race/ethnicity			0.51 (0.48)		
White	627 (78.4)	143 (80.8)			
Non-white	173 (21.6)	34 (19.2)			
Education			4.37 (0.11)		
High school	283 (35.4)	51 (29.1)			
Some college	138 (17.3)	26 (14.9)			
College	378 (47.3)	98 (56.0)			

Bold indicates significance at alpha level of 0.05.

*Note*: Frequencies not summing to *N*=981 and percentages not summing to 100 reflect missing data.

BMI, body mass index; LGBTQ, lesbian, gay, bisexual, transgender, and queer; SD, standard deviation.

**Table 2.** Health Characteristics of Older Deaf Lesbian, Gay, Bisexual, Transgender, and Queer (LGBTQ) and Non-LGBTQ Adults

	Non-LGBTQ (*n*=803)	LGBTQ (*n*=178)	*χ^2^* (*p*-value)
	*n* (%)	*n* (%)	
Health status			0.33 (0.96)
Poor/fair	86 (11.0)	17 (9.7)	
Good	300 (38.3)	66 (37.7)	
Very good	272 (34.7)	63 (36.0)	
Excellent	125 (16.0)	29 (16.6)	
Health insurance coverage			4.94 (0.09)
No	18 (2.3)	9 (5.2)	
Yes	761 (97.1)	162 (93.6)	
Don't know	5 (0.6)	2 (1.2)	
Regular provider 1.03 (0.31)			
No	173 (29.6)	34 (25.2)	
Yes	412 (70.4)	101 (74.8)	
Diabetes			0.002 (0.96)
No	580 (73.7)	125 (73.5)	
Yes	207 (26.3)	45 (26.5)	
Hypertension			1.65 (0.20)
No	382 (48.7)	93 (54.1)	
Yes	403 (51.3)	79 (45.9)	
Heart condition			0.85 (0.36)
No	672 (85.6)	151 (88.3)	
Yes	113 (14.4)	20 (11.7)	
Chronic lung disease/asthma/emphysema/chronic bronchitis			**7.09 (0.01)**
No	661 (85.0)	131 (76.6)	
Yes	117 (15.0)	40 (23.4)	
Arthritis/rheumatism			0.63 (0.43)
No	471 (59.9)	98 (56.6)	
Yes	315 (40.1)	75 (43.4)	
Depression/anxiety disorder			**20.37 (<0.001)**
No	642 (82.1)	114 (66.7)	
Yes	140 (17.9)	57 (33.3)	
Stroke			0.66 (0.42)
No	709 (95.6)	159 (94.1)	
Yes	33 (4.4)	10 (5.9)	
Cirrhosis/liver/kidney problems			0.67 (0.41)
No	672 (90.8)	150 (88.8)	
Yes	68 (9.2)	19 (11.2)	
Cancer			**6.39 (0.05)**
No	585 (75.9)	145 (84.8)	
Yes	186 (24.1)	26 (15.2)	
Comorbidity (2 or more diagnoses)			2.96 (0.09)
No	340 (44.2)	64 (37.0)	
Yes	430 (55.8)	109 (63.0)	

Bold indicates significance at alpha level of 0.05.

*Note*: Frequencies not summing to *N*=981 and percentages not summing to 100 reflect missing data.

As shown in Table 2, between Deaf LGBTQ and non-LGBTQ groups, significant differences emerged for several medical conditions. In particular, Deaf LGBTQ participants reported significantly higher proportions of chronic lung disease/asthma/emphysema/chronic bronchitis, depression/anxiety, and personal cancer history compared with Deaf non-LGBTQ participants. After controlling for known correlates of medical conditions (sex, education, age, race, and health status), self-identication as LGBTQ was significantly associated with increased risks for lung disease/asthma/emphysema/chronic bronchitis, arthritis, depression/anxiety, and comorbidity (Table 3).

**Table 3.** Relative Risk Ratio Estimates for Medical Conditions by Lesbian, Gay, Bisexual, Transgender, and Queer Status

Medical condition	LGBTQ (ref: non-LGBTQ)	
	RR	95% CI
Diabetes	1.21	0.92–1.60
Hypertension	0.99	0.83–1.18
Heart condition	0.96	0.66–1.57
Lung disease/asthma	**1.74**	**1.26–2.42**
Cancer	0.75	0.51–1.10
Arthritis	**1.26**	**1.05–1.53**
Stroke	1.50	0.72–3.15
Cirrhosis/kidney/liver problems	1.39	0.85–2.29
Depression/anxiety disorder	**1.71**	**1.30–2.25**
Comorbidity (2 or more medical conditions)	**1.25**	**1.10–1.43**

Values in bold indicate statistical significance. Adjusted for age, sex, race, education, and health status.

CI, confidence interval; RR, relative risk.

## Discussion

While no census data are available on the number of older Americans identifying as LGBTQ, estimates have suggested their number lies in the range of 1.75 to 4 million adults.^18^ That number is increasing, and the number of LGBTQ Americans over age 50 years is expected to reach 5 million by 2030.^18^ The growing number of young Americans who openly identify as LGBTQ^19^ may align with a corresponding future increase in the American population of LGBTQ mid-to-older adults, including those who are Deaf and use ASL. Accordingly, medical and psychological service providers will need to be aware and informed about the health inequity issues facing mid-to-older Deaf LGBTQ Americans, to provide culturally competent and linguistically accessible health care for Deaf LGBTQ patients.

To date, no research has explored health disparities among Deaf adults who self-identify as LGBTQ and those who do not. Thus, this study is among the first of its kind to examine disparities in medical conditions among Deaf LGBTQ and Deaf non-LGBTQ adults who use ASL. Consistent with the health disparities in the hearing LGBTQ and non-LGBTQ general population, findings from our study do indicate within-group health disparity between Deaf LGBTQ and non-LGBTQ adults. In particular, the relative risks for lung diseases, arthritis, depression or anxiety, and comorbidity are found to be higher for Deaf LGBTQ adults than Deaf non-LGBTQ adults.

The higher prevalence of chronic lung-related health issues reported by Deaf LGBTQ participants compared with Deaf non-LGBTQ participants is consistent with similarly higher rates of lung and chronic obstructive lung diseases found among the national LGBT adult population.^20^ Notably, the CDC found that 23.9% of lesbian, gay, and bisexual adults in the United States reported histories of cigarette smoking compared with 16.6% of heterosexual adults.^21^ This finding may suggest that similar smoking-related risk factors are experienced by Deaf LGBTQ adults compared with Deaf non-LGBTQ adults. For example, related risk factors may include smoking to manage daily stresses associated with anti-LGBTQ stigma and discriminatory encounters^22^; higher rates of secondhand smoke exposure (given LGBTQ-friendly bars as historically safe spaces and thus venues frequently designated for tobacco interventions)^23^; and targeted marketing by tobacco companies that often sponsor large-scale LGBTQ-friendly events, programs, and festivals. Because such events frequently retain ASL interpreters and provide accessible opportunities for social networking, Deaf LGBTQ consumers may be likely to attend.

Among the general population of lesbian, gay, and bisexual adults with depression or anxiety, associated contributing factors may include experiences of victimization, internalized stigma, financial barriers to health care, and poor physical health.^24–26^ Due to scarcity of mental health research among Deaf LGBTQ populations, additional studies are needed to examine affiliation with, and social support received through, Deaf and signing communities as a potential resiliency factor mitigating depression and anxiety risk.

Of note, barriers to psychological help-seeking among hearing LGBTQ patients may include societal stigmas, adverse coping behaviors, challenges in accessing insurance, and gaps in culturally specific knowledge related to mental health and illness. For many, obstacles may include outright discrimination and lack of provider sensitivity^27^; subpar provider treatment, ill-equipped and inadequate facilities, and exclusionary office climates.^28^ Deaf LGBTQ patients may face similar challenges. However, when seeking help for mental health-related concerns, Deaf LGBTQ patients may be required to manage additionally complex processes of determining relative safety around disclosure of SOGI information to health care providers, potentially as providers may be unfamiliar both with communication needs of Deaf individuals and culturally competent practices for working with LGBTQ patients. Further, mental health disparity between Deaf LGBTQ and non-LGBTQ patients may be perpetuated through systemic inequities and considerable challenges to accessing adequate, timely, and culturally informed mental health education and care, rendering help-seeking an especially precipitous task for Deaf LGBTQ patients.

The results of the current study suggest that heterosexual patients self-report higher rates of cancer; however, this is in contrast to a previous study.^29^ Additionally, a 2017 study of 536 older adults found that marginalization and stigma were associated with higher rates of cancer reported among sexual minority older men compared with heterosexual older men.^30^ Interestingly, among Deaf LGBTQ adults, those who self-identified as female were significantly less likely to disclose SOGI-related information to their health care providers compared with those who self-identified as male.^11^ The authors propose that these disparate rates may be due to missed cancer diagnoses. That is, as LGBTQ patients frequently experience increased stigma related to SOGI, preventive and first-line health care may be even further postponed among Deaf individuals considering communication barriers. This may have a particular downstream impact on Deaf LGBTQ female patients uncomfortable with disclosing sexual orientation and/or transgender identities to their providers, possibly contributing to underreported symptoms and delayed or missed diagnoses.^31,32^ Future research is needed to replicate the results in other samples to clarify the aspects and influences of intersectional identities for cancer health disparities within the Deaf population.

The higher relative risks for arthritis and comorbidity among Deaf LGBTQ individuals compared with Deaf non-LGBTQ individuals are consistent with the general literature indicating similar risks in hearing lesbian, gay, or bisexual adults aged 50 years or older.^33^ The review of the literature in this National Health, Aging, and Sexuality/Gender Study study also found that the LGBTQ group, compared with non-LGBTQ hearing adults, were also more likely to report poor general health, mental distress, and smoking habits. Arthritic conditions may involve multiple organs resulting in varied and widespread symptoms, and as such may be linked to metabolic, genetic, infectious, traumatic, and immunological factors. Among those with arthritis, the challenges of managing multiple medications and resulting drug interactions, impacts on cognitive function, and limitations on mobility and activities of daily living may exacerbate mood-related symptoms and overall constrict access to social and economic supports. Older LGBTQ adults, particularly those who are Deaf or hard of hearing, may be less apt to follow through on routine health care appointments or engage in preventive self-care behaviors due to communication barriers and challenges to health literacy.

### Strengths and limitations

A major strength of this study is its inclusion of the largest U.S. sample of mid-to-older deaf, ASL-using LGBTQ-identified adults to date. A limitation is that the study did not assess risk factors in relation to intersectional identities due to insufficient number of responses when broken down by medical conditions, ethnic/racial group, and LGBTQ status; such analysis could have provided further research insights into disparities particularly unique to subgroups of Deaf LGBTQ older adults.

### Health equity implications

The mental and physical health disparities experienced by Deaf LGBTQ communities may be better understood in the context of stressors impacting physical and mental health, systemic and individually encountered barriers to health knowledge, and societal stigmas associated with these psychosocial and sociocultural identities. Providers working with Deaf LGBTQ patients—who as a population increasingly comprise older adults—should strive to provide equitable, culturally competent, and linguistically accessible services with particular attention to patient risk factors that may be associated with physical and mental health-related conditions.

Like their hearing LGBTQ counterparts, Deaf LGBTQ mid-to-older adults may require more frequent, comprehensive, or uniquely tailored health care services and social support. Need for such services and support may be particularly demanded given risk factors related to and arising from anti-LGBTQ stigma and low rates of provider cultural and linguistic competency. Culturally and linguistically competent care by health care providers at all levels is invaluable in promoting health equity, particularly when provider education and training is undergirded by an intersectional framework that examines and treats based on the interaction and context of multiple patient and provider social identities.

## Abbreviations Used

ASL: American Sign Language
BMI: body mass index
HINTS: Health Information National Trends Survey
LGBTQ: lesbian, gay, bisexual, transgender, and queer
RR: relative risk
SD: standard deviation
SOGIs: sexual orientation and/or gender identities
